# FV peptide induces apoptosis in HEp 2 and HeLa cells: an insight into the mechanism of induction

**DOI:** 10.1186/1477-3163-5-27

**Published:** 2006-12-01

**Authors:** M Sri Balasubashini, S Karthigayan, ST Somasundaram, T Balasubramanian, R Rukkumani, Venugopal P Menon

**Affiliations:** 1 Department of Biochemistry, Annamalai University, Annamalai Nagar, 608 002, India; 2 CAS in Marine Biology, Annamalai University, Parangipettai, 608 502, India; 3 Department of Biochemistry, Center for Cell and Molecular Biology, Hyderabad, India

## Abstract

The present study is an attempt to evaluate the antiproliferative potential of peptide (7.6 kDa) from lionfish (*Pterios volitans*) venom on cultured HEp2 and HeLa cells. Different dose of purified peptide (1, 2 and 4 μg/ml) at different time points (12, 24 and 36 hrs) were tested for antiproliferative index of the peptide. Among them, 2 μg/ml at 24 hrs was found to effectively inhibit cancer cell growth *in vitro *and did not cause any adverse effect on normal human lymphocytes. Apoptosis was examined by propidium iodide staining, confirmed by the expression of caspase-8 and caspase-3, down regulation of Bcl-2 expression and DNA fragmentation in treated cells, when compared to untreated HEp2 and HeLa cells. Thus fish venom peptide was found to selectively induce apoptosis in cancer cell.

## Background

Cancer is the major public health burden in all developed countries. Currently one in four death in the United States is due to cancer [1]. In all types of cancer, genetic alterations give rise to changes in expression, activation or localization of regulatory proteins in the cells. They then affect the signaling pathways that alter their response to regulatory stimuli and allow the unrestricted cell growth. Chemotherapeutic treatment strategies attempt directly to inhibit proliferation of cancer cells or selectively remove transformed cells by inducing apoptosis or eliminating the cause of the growth advantage [2].

The approach of testing venoms as antitumour agents dates back to the beginning of the last century, when Calmette et al., [3] reported on the antitumour activity of snake venom, (Naja species venom) in adenocarcinoma cells. Since then many anticancer compounds have been isolated from toxins of marine sponges e.g. Sesterstatin, Dolastatin 10, Crellastatin and Ecteinascidin 743 from tunicate is under Phase II clinical trial [4]. It was also demonstrated that purified protein from cobra venom was selectively cytotoxic to cancer cells [5,6]. Particularly, proteinacious venom from several animals like snake, scorpion, spider etc., was reported to have excellent cytotoxicity in cultured cancer cell lines and also reduces tumor growth in mice [7,8]. Among all, snake venom has been studied extensively however, the marked curative properties of the snake venoms are always hindered by their high toxicities, and hence we selected a less toxic species, scorpion fish venom for the present study.

*Pterios volitans *belongs to the family Scorpaenidae and inhabits coral reef of the Indo-Pacific region and the Red sea [9]. It is a relative of both stonefish (*G.marmoratus*) and soldier fish (*S.trachynis*). *P.volitans *venom apparatus consist of dorsal, anal and pelvic spines. Symptoms of *P.volitans *envenomation resemble those of other fish of family Scorpaenidae, in particular edema and extreme pain associated with the site of injury. Systemic effects such as vomiting, fever and sweating have been reported [10]. Previous studies on *P.volitans *venom have observed negative inotropic and chronotropic effects *in vitro *[11] and depressor effects *in vivo *[12]. Previously we have reported that lionfish venom reduces the tumour burden and inhibits the metastasis in *In vivo *animal model [13]. However, the role of active component of the venom, its molecular target and signaling pathways through which it cause apoptosis in cancer cells is unknown.

But, unlike cancer cells, normal cells have intact programmed cell death mechanisms and most treatments designed to kill cancer cells also reach normal cell, resulting in side effects to organs including the gastrointestinal tract, bone marrow etc., The aim of the present study was to purify the active peptide in the fish venom and to investigate its antiproliferative effect on cultured HEp2, HeLa and normal lymphocytes *In vitro *and thereby its possible mechanism of action.

## Experimental procedures

### Chemicals and reagents

The HEp2 (alveolar epithelial carcinoma cell line) and HeLa (Cervial cancer line) cell lines were obtained from National Center for Cell Science, Pune. All fine chemicals were obtained from Sigma-Aldrich, St. Louis, MO, USA. [^3^H]thymidine was from Amersham, UK. Bcl-2 antibody was obtained from Santa Cruz Biotechnology, Inc. Santa Cruz, California, Caspase-3 and 8 antibodies were obtained from BD PharMingen, USA.

### Venom preparation

Specimens of *P.volitans *were obtained from the local aquarium, killed (by cooling) and the venomous spines were removed and stored in 10% glycerol solution at -80°C and the venom was prepared as described by Church and Hodgson [9]. The protein was estimated by Lowry et al., [14]. The concentration was adjusted to 1.0 mg/ml, aliquoted and stored at -20°C till use.

### Purification and MALDI-TOF analysis

The active crude fish venom was applied to sephadex G-50 column, equilibrated with 0.1 M phosphate buffer (potassium dihydrogen phosphate and dipotassuim hydrogen phosphate pH 7.4) and the fractions collected were checked for activity on the HEp2 and HeLa cell lines. The active fractions were pooled and the further concentrated using a 10 kDa Amicon filter (Millipore corporation, Billerica, MA, USA). The activity was once again determined with the cell lines and run on the SDS-PAGE. Again the purity was checked by the reverse phase HPLC using C18 column. A single peak with the retention time of 25 min was obtained. Purification was carried out in three batches under same experimental condition. The yield of the pure peptide was 160 μg/ml and was pooled together to carryout the experiments.

Thus purified sample was submitted to Matrix-Assisted Laser Desorption/Ionization (MALDI) Time-of-Flight (TOF) mass spectrometry analysis using a Voyager-DE PRO equipment (PerSeptive Biosystems) under the following conditions: accelerated voltage 25 kV, laser Wxed at 2890_J/com2, and delay 300 ns, in the linear analysis mode. Samples (10–50 pmol) were dissolved in 0.1% (v:v) TFA in water and applied onto a target to determine the molecular weight of the peptide.

### *in vitro *antiproliferative assay using [^3^H]thymidine incorporation

HEp2 and HeLa cell lines were maintained in F-12 Dulbecco's Modified Eagle Meduim (DMEM) supplemented with 10% Fetal calf serum, amphotericin (3 μg/ml), gentamycin (400 μg/ml), streptomycin (250 μg/ml), penicillin (250 units/ml) in a carbon dioxide incubator at 5% CO_2_.

In a 24 well plate [^3^H]thymidine (1 μci/ml of medium) was added to the medium in which the cell line was already maintained. 20 μl of different concentration (1, 2, 4 and 8 μg/ml) of the fish venom (FV) peptide was added to the cells. Same volume of buffer was added to the control well. The cultures were trypsinized at the desired time points, pelleted and washed sequentially with 10% and 5% tricholoroacetic acid and solublized in 0.1% sodium hydroxide and 0.025% sodium dodecyl sulphate solution. The radioactivity of the samples was measured in the Packard, TopCount. NXT™ Liquid scintillation counter and expressed as cpm/mg protein. From the above experiment an appropriate dose is selected and added to human lymphocytes (isolated using histopaque – Sigma, USA) to check the effect on normal cells.

### MTT assay for cytotoxicity

HEp2 and HeLa cells were incubated in 96 wells plate (1 × 10^6 ^cells/ml) in the presence and absence of FV peptide (2 μg/ml) for different time points (0, 12, 24, 36, 72 and 96 h). At the required time point 50 μl of supernatant was aspirated, added to another well and mixed with 50 μl of the substrate buffer containing MTT dye (provided in the Promega kit). The mixture was incubated for 30 minutes at 15 to 25°C. Care was taken to keep the samples protected from light. After 30 minutes incubation 50 μl of the stop solution was added and the absorbance was read on an ELISA reader at 490 nm.

Raw absorbance readings were used to calculate the percentage cell death compared to that released by triton X-100 detergent. Percent cell death was calculated as [(values from experimental condition) - (values from untreated control)]/[(values from triton treated wells) - (values from untreated control)] × 100. Each experiment was performed at least 6 times to ensure consistency.

## Detection of apoptosis

### Propidium Iodide staining

HEp2 and HeLa cells were grown in a 6 well plate, FV peptide and control drug were added and incubated for 24 hrs and then cells were trypsinised and collected in micro-centrifuge tubes. Cells were resuspended in 50 μl of PBS. 5 μl of RNase (1 mg/ml) and 5 μl of Propidium Iodide (25 μg/ml in PBS) was added and incubated at 37°C for one hour. Fluorescence was excited with an Argon ion laser at 488 nm and visualized under Nikon Fluorescence microscope.

### Immunoblot analysis

Total cell lysate were prepared as reported by Narayana et al., [15]. Proteins were separated on SDS 10% polyacrylamide gel. The gel was transferred onto a nitrocellulose membrane (hybond C+, Amersham Life Sciences) at 220 mA for 3 h. The membrane was then washed with PBS and blocking reagent (3% skimmed milk) was added and blocked overnight at 4°C. Blocking reagent was washed with PBS twice for 5 min each and primary antibody was added (Bcl-2, caspase 3 and 8). One percent BSA in PBS, 0.1% Tween 20 was added to the blot along with the primary antibody (1:2000) and rocked gently at room temperature for 1 h. The blot was washed three times with PBS for 5 min each. Secondary antibody (1:5000) in 1% BSA in PBS, 0.1% Tween 20 was allowed to hybridize for 1 h at room temperature. The bands were detected using the chromogenic substrate NBT-BCIP in alkaline phosphatase buffer.

### DNA fragmentation assay

Internucleosomal cleavage of DNA was analysed as given below. Briefly, cells were treated with FV peptide (2 μg/ml). The plate was incubated for required time points. Cells were washed twice with ice-cold PBS and resuspended in lysis buffer containing 10 mM Tris – HCl (pH 8.0), 20 mM EDTA and 0.5% Triton X-100 and incubated for 30 min in ice. After centrifugation at 10000 rpm for 10 min at 4°C, DNA was extracted with phenol chloroform, precipitated with 0.1 vol 3 M sodium acetate and 2.5 vol ethanol and stored at -20°C for overnight. DNA was pelleted by centrifugation at 10,000 rpm for 5 min at 4°C, rinsed with 70% ethanol and then resuspended in TE buffer (pH 8.0) containing, 30 μg/ml of RNase and incubated for 6 hr at 37°C. DNA was run on a 2% agarose gel with ethidium bromide staining [16]. After electrophoresis, DNA was visualized under UV and documented.

### Statistical analysis

Values are expressed as means ± S.D of 6 repeats in each group. Data within the groups were analyzed using one-way analysis of variance (ANOVA) followed by Duncan's Multiple Range Test (DMRT). A value of P < 0.05 was considered statistically significant.

## Results

### Purification of active peptide

**Figure 1:**
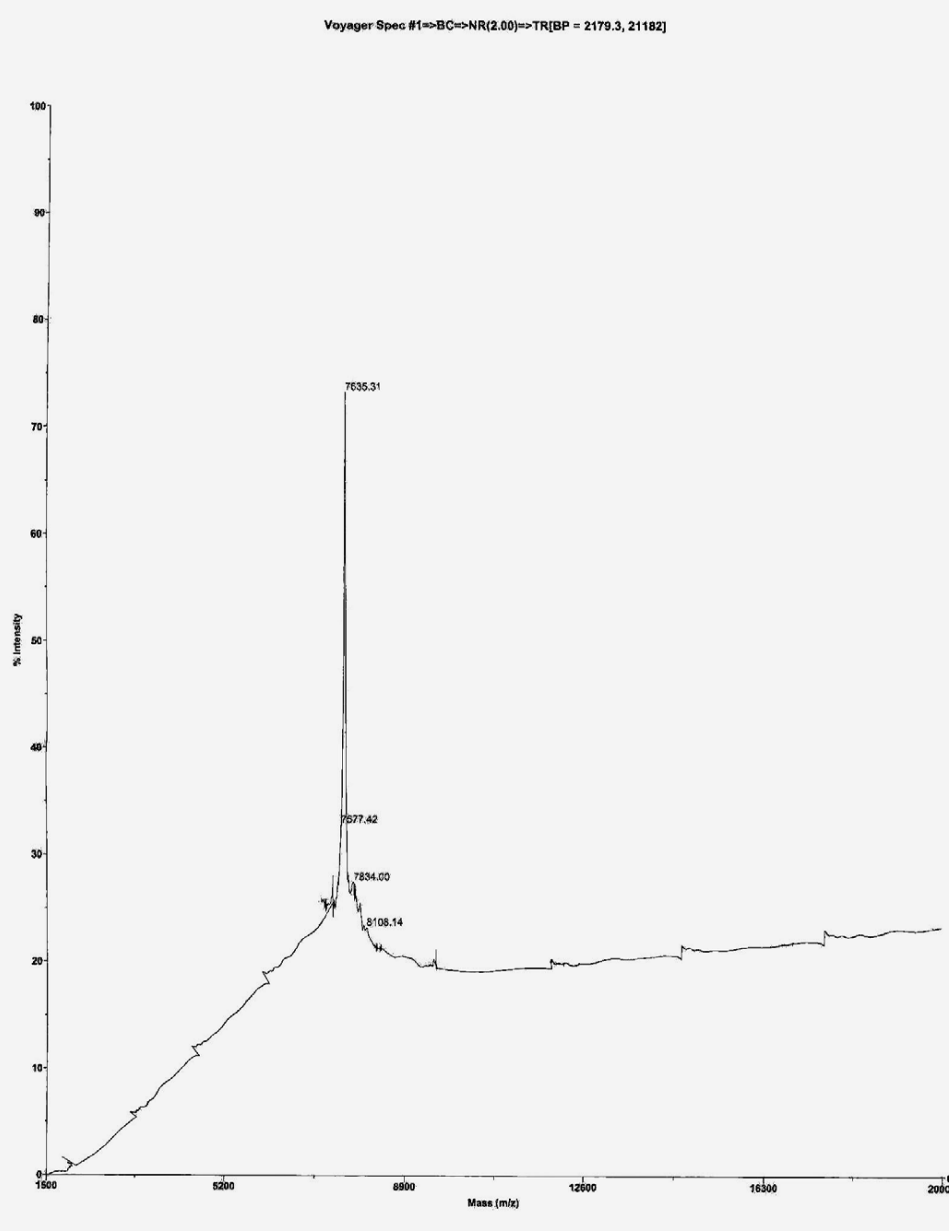
MALDI-TOF spectrum showing the molecular weight of the purified FV peptide.

MALDI-TOF spectrum showing the molecular weight of purified peptide from the crude fish venom is illustrated in the Fig. 1. This spectrum shows the presence of a single intense peak with a molecular weight of 7.6 kDa. Thus purified peptide was checked for its activity and used for the further analysis.

### Antiproliferative studies

**Figure 2:**
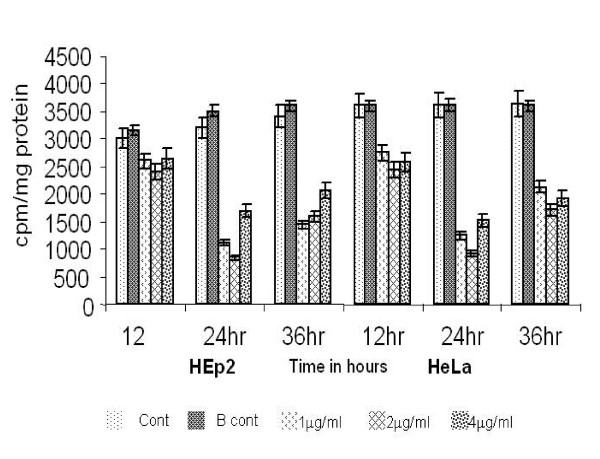
Antiproliferative effect of FV peptide on HEp2 and HeLa cell lines.

The preliminary antiproliferative effect of fish venom (FV) peptide on HEp2 and HeLa cell lines are shown in Fig.2. [^3^H]thymidine incorporation studies were conducted in three time points 12, 24 and 36 h. There was marked inhibition of proliferation by FV at 24 h. The results are expressed as counts of [^3^H]thymidine incorporated into the actively proliferating cells per min per mg protein of the cell mass. In the Fig. 2 the cell control and PBS control shows the maximum counts of [^3^H]thymidine (around 36000 cpm/mg protein) when compared to the treatment groups. Dose dependent study shows that 2 μg/ml of FV showed the maximum inhibition (8500 and 9100 cpm/mg protein for HEp2 and HeLa cells respectively) and this same dose (2 μg/ml) did not show any cytotoxicity to the normal human lymphocytes.

### MTT assay for cytotoxicity

**Figure 3:**
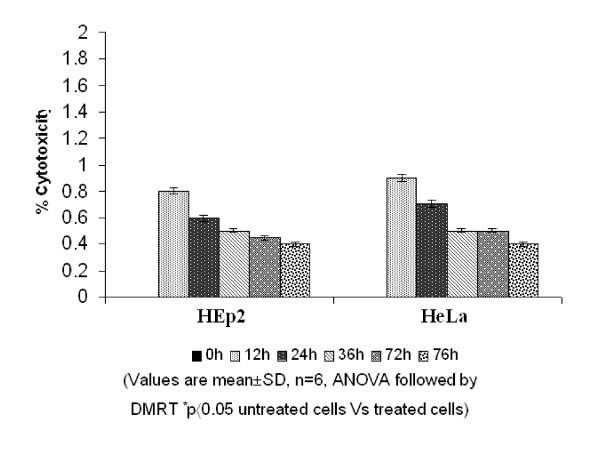
MTT assay showing the cytotoxicity of FV peptide.

The cytotoxic effect was assed by the release of lactate dehydrogenase (LDH) from HEp2 and HeLa cells after treating with FV peptide at various time points did not exceed 0.8% and suggest that the antiproliferative effect is by induction of apoptosis in treated cells (Fig. 3). Hence this dose alone was used for further studies.

### Propidium Iodide staining to study nuclear morphology

**Figure 4:**
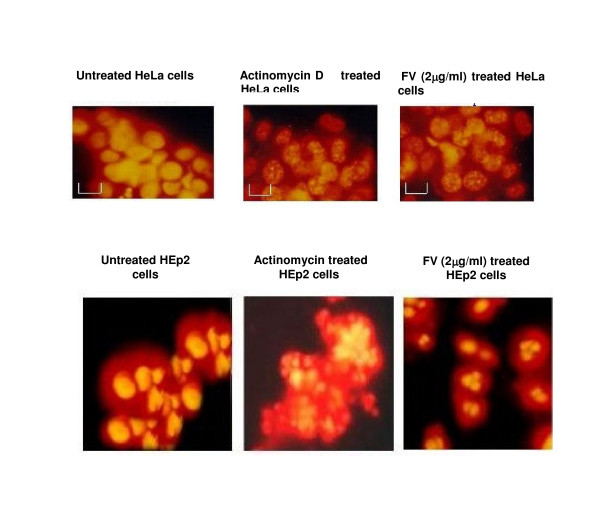
**shows the nuclear localization of HEp2 & HeLa cells by propidium iodide staining**. a and d are untreated HeLa and HEp2 cells respectively showing the intact cells without any nuclear fragmentation. b and e are actinomycin treated positive control. c and f are FV (2 μg/ml) treated cells showing the characteristic nuclear fragmentation and apoptotic bodies when compared to the untreated cells.

Monolayer of HEp2 and HeLa cells were treated with FV (2 μg/ml) and the extent of apoptosis was assessed by propidium iodide staining. The treated monolayer of HEp2 and HeLa cells contained more apoptotic cells when compared to untreated monolayer (Fig. 4). There was characteristic nuclear fragmentation of nuclei in treated HEp2 and HeLa cells whereas; the untreated control cells did not show any nuclear fragmentation. The apoptotic cells displayed the characteristic features of reduced size, intense fluorescence of condensed nuclear chromatin and formation of membrane blebs. Similar results were observed with same dose of venom extract on the HeLa cells.

### Analysis of Caspase – 8, Caspase – 3 and Bcl-2 protein expression in HEp-2 cells

**Figure 5:**
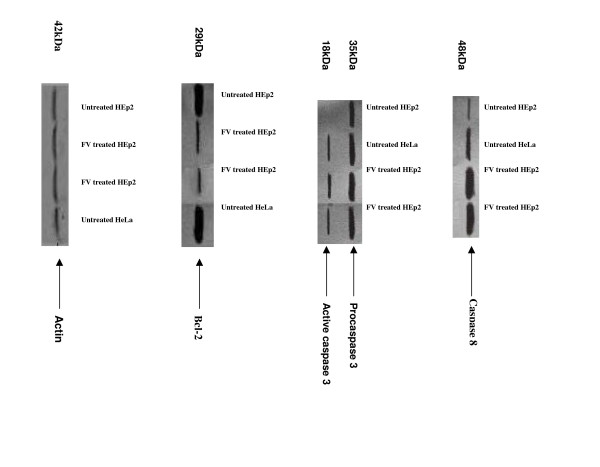
**shows the immunoblot of Caspase 3, 8 & Bcl-2 expression in untreated and treated HEp2 & HeLa cells. A. Expression of caspase 8**. Lane 1 & 2 : untreated HEp2 and HeLa cells. Lane 3 & 4 : HEp2 & HeLa treated with FV (2 μg/ml). **B. Expression of pro and active caspase 3**. Lane1 & 2 : untreated HEp2 and HeLa cells. Lane 3 & 4 : HEp2 & HeLa treated with FV (2 μg/ml). **C. Expression of Bcl -2**. Lane 1 & 4 : untreated HEp2 and HeLa cells. Lane 2 & 3 : HEp2 & HeLa treated with FV (2 μg/ml). **D. Expression of actin (loading control)**. Lane 1 & 4 : untreated HEp2 and HeLa cells. Lane 2 & 3 : HEp2 & HeLa treated with FV (2 μg/ml)

Immunoblot analysis of Caspase-8 and Caspase-3 in HEp-2 and HeLa cells on exposure to FV (2 μg/ml) for 24 h showed activation of Caspase-8 in treated cells (Fig 5A) when compared to the untreated control cells. The activated caspase-8 in turn cleaves the pro caspase-3 (32 kDa) into active Caspase-3 (17 kDa) (Fig. 5B). This is further confirmed by the down regulation of Bcl-2 expression in treated cells, which is actively expressed in the untreated cells (Fig. 5C).

### DNA fragmentation

**Figure 6:**
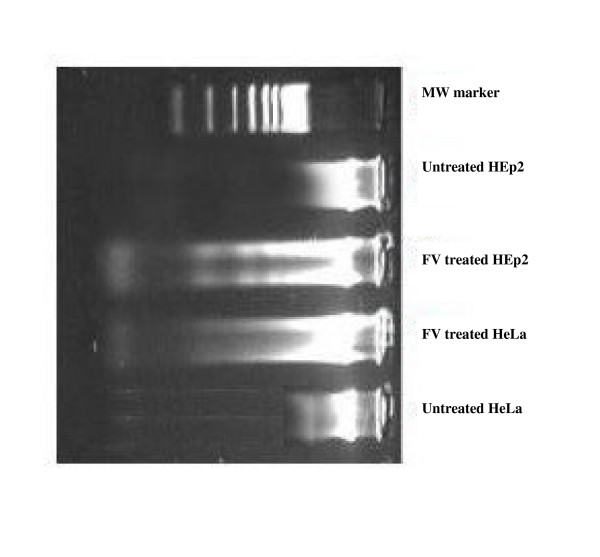
**Shows the DNA fragmentation analysis**. Lane 1 : Molecular weight marker. Lane 2 & 5 : Untreated HEp2 and HeLa cells. Lane 3 &4 : FV (2 μg/ml) treated HEp2 and HeLa cells

Fig. 6 shows the agarose gel electrophoresis of chromosomal DNA isolated from the HEp2 and HeLa cells treated with FV (2 μg/ml), along with untreated control. Lane 1 shows the marker, 100 bp DNA ladder. Lane 2 and 5 shows the intact DNA of the untreated HEp2 and HeLa cells, when compared to the fragmented DNA (HEp2 and HeLa cells) of treated cells on Lane 3 and 4 respectively. This study clearly demonstrates the DNA damage caused by the fish venom extract when compared to the untreated cells.

## Discussion

Cancer cell cultures have been useful models to evaluate gene expression and to establish *in vitro *experiments aiming at the control of tumor cell growth [17]. One of the major defects in cancer is the lack of cells to be driven into the apoptotic mode, due to malfunction of molecules like c-myc [18], ras [19], p53 [20], Bcl-2 [21], caspases [22] and telomerase [23]. Hence we thought that, targeting such molecules in cancer might provide a new therapeutic strategy. With this background we tired to evaluate the antiproliferative effect of fish venom (FV) peptide and induction of apoptosis in cultured HEp2 and HeLa cells.

The antiproliferative effect was first evaluated by [^3^H]thymidine uptake of the actively proliferating cells. Among the different doses tested at different time points, 2 μg/ml at 24 h was found to produce a marked inhibition on the HEp2 and HeLa cell proliferation and this dose did not show any adverse effect on normal human lymphocytes. At longer time points, 36 h, the antiproliferative effect was reduced, which may be due to the effect of temperature on the venom and needs to be investigated further. However, fish venom was found to have selective sensitivity to cancer cells. Here, the question arises, whether the antiproliferative effect is by apoptotic or necrotic pathway. This was confirmed by MTT assay, the release of LDH is considered to be of necrotic pathway and the treated cells showing very less levels of LDH after 24 h suggests that the event of cell death is by apoptosis. Lipps observed similar results for the two protein fractions from snake venom, Atroporin and Kaotree on SP/2 cell line [24]. Moreover, Greel and Vaux [25] have suggested that a cancer cell treated with a potentially lethal toxin will not die because of the direct effects of toxin, but will activate its suicide mechanism to die earlier by apoptosis. Thus in light of the previous findings it is evident that FV induces apoptosis selectively in cancer cells.

The role of apoptosis in cancer has been studied in detail. Apoptosis being a highly regulated cellular response with crucial checkpoints regulates the fate of cells. These checkpoints are processing centers sensing extra cellular signals, amplifying localized signals, integrating information form these cells and directing them towards death cascade [26]. One of the targets of oncogene-induced sensitization is the mitochondria and c-myc facilitates cytochrome c release from the mitochondria [27]. The released cytochrome c activates the caspases, a family of cysteine protease and suppresses Bcl-2 thereby causing apoptosis through intrinsic pathway [28].

The possible role of apoptosis in the present study was first examined with propidium iodide staining of treated and untreated cells. The nuclear fragmentation (fig. 5) clearly demonstrates the role of apoptosis in FV treated cells and motivated to further analyze the expression of certain apoptotic markers. Expression of caspase-8 was observed in the FV treated cells when compared to untreated cells, which in turn was believed to activate the procaspase-3 to active caspase-3 [29]. FV treatment was also found to down regulate the expression of Bcl-2 when compared to cancer control. Hence it is obvious that FV peptide selectively targets the cancer cells sparing the normal ones.

The caspase-activated cascade finally results in the DNA fragmentation. Willey has suggested that, during apoptosis a specific nuclease cuts the genomic DNA between nucleosomes to generate DNA fragments, and the presence of this ladder has been extensively used as a marker for apoptotic cell death [30]. Nagata also showed that, the DNA ladder nuclease (now known as caspase activated DNase or CAD) pre-exists in living cells as an inactive complex with an inhibitor subunit. Activation of CAD occurs by means of caspase-3-mediated cleavage of inhibitory subunit, resulting in the release and activation of the catalytic subunit [31]. The observed result, activation of caspases and DNA fragmentation demonstrates the role of FV in apoptosis of HEp2 cells.

Till date many toxins and proteins have been reported to induce apoptosis in the cancer cell by activating the enzymes of the apoptotic cascade [32,33]. Previously, we have reported that lionfish venom induces apoptosis in Ehrlich's ascites carcinoma xenografted mice by activating caspase-3, which was evidenced by nuclear fragmentation [13]. In this context, Snyder et al., [34] has suggested a hypothesis of anti-cancer gene therapy, which says that, replacement of tumor suppressor gene functions in malignant cell will result in specific death or apoptosis of the cancer cell, while sparing the surrounding normal cells. We have reported the anticancer effect of fish venom for the first time in the *In vitro *cancer model.

Hence the present study clearly demonstrate that FV peptide targets the actively proliferating cancer cells by inducing the apoptotic pathway and mechanism by which the induction is made is yet to be resolved.
